# Information Quality in Regulatory Decision Making: Peer Review versus Good Laboratory Practice

**DOI:** 10.1289/ehp.1104277

**Published:** 2012-02-17

**Authors:** Lynn S. McCarty, Christopher J. Borgert, Ellen M. Mihaich

**Affiliations:** 1 L.S. McCarty Scientific Research & Consulting, Newmarket, Ontario, Canada; 2 Applied Pharmacology and Toxicology, Inc., Gainesville, Florida, USA; 3 Center for Environmental and Human Toxicology, Department of Physiological Sciences, University of Florida College of Veterinary Medicine, Gainesville, Florida, USA; 4 Environmental and Regulatory Resources, Durham, North Carolina, USA

**Keywords:** data quality, GLP, peer review, regulatory decision making, toxicity tests

## Abstract

Background: There is an ongoing discussion on the provenance of toxicity testing data regarding how best to ensure its validity and credibility. A central argument is whether journal peer-review procedures are superior to Good Laboratory Practice (GLP) standards employed for compliance with regulatory mandates.

Objective: We sought to evaluate the rationale for regulatory decision making based on peer-review procedures versus GLP standards.

Method: We examined pertinent published literature regarding how scientific data quality and validity are evaluated for peer review, GLP compliance, and development of regulations.

Discussion: Some contend that peer review is a coherent, consistent evaluative procedure providing quality control for experimental data generation, analysis, and reporting sufficient to reliably establish relative merit, whereas GLP is seen as merely a tracking process designed to thwart investigator corruption. This view is not supported by published analyses pointing to subjectivity and variability in peer-review processes. Although GLP is not designed to establish relative merit, it is an internationally accepted quality assurance, quality control method for documenting experimental conduct and data.

Conclusions: Neither process is completely sufficient for establishing relative scientific soundness. However, changes occurring both in peer-review processes and in regulatory guidance resulting in clearer, more transparent communication of scientific information point to an emerging convergence in ensuring information quality. The solution to determining relative merit lies in developing a well-documented, generally accepted weight-of-evidence scheme to evaluate both peer-reviewed and GLP information used in regulatory decision making where both merit and specific relevance inform the process.

The validity and credibility of scientific data is central to all scientific endeavors, as well as to decision structures that use such data (Schreider et al. 2010). Principal among those are risk assessments, safety assessments, and regulatory decisions routinely made by federal agencies such as the U.S. Environmental Protection Agency (EPA), the Food and Drug Administration (FDA), and the U.S. Department of Agriculture (USDA) in the United States or in similar agencies in other jurisdictions. Regulatory decisions are often questioned because either the type or the source of the data relied upon comes under scrutiny. Regulatory decisions have been challenged for relying on data that allegedly lack relevance or sensitivity for the protection of public health and the environment and for relying on data generated by scientists or laboratories perceived to have a conflict of interest regarding the outcome of the decision (e.g., Myers et al. 2009). Some proposed solutions argue for transparency and stress the availability of raw data and methodological details as the principal means of enhancing credibility (Borgert 2007; Schreider et al. 2010).

More transparency may increase the credibility of decisions because it enhances the perceived honesty of the process. On the other hand, transparency and honesty, in and of themselves, do not address underlying questions about data quality. Peer-review requirements for scientific journals and data acceptance requirements for regulatory programs both acknowledge that a rigorous evaluation of data quality is essential, yet the practices and procedures for addressing it differ across the spectrum of bodies that deal with scientific data. These differences may arise from disparate definitions of data quality but more likely relate to the reasons for adjudicating data quality, which differ according to the purview of these bodies. In this review, we compare and contrast different operational definitions of data quality assumed for regulatory acceptance and for publication in peer-reviewed journals. We then analyze how these different standards apply to regulatory decisions in environmental health and safety, with emphasis on how each may contribute to or detract from the credibility of the decision-making process. To illustrate the issues involved, we refer to the current debate on this topic in endocrine disruptor research.

The concept of “endocrine disruptors” or “hormonally active agents” (National Research Council 1999) has been debated strenuously since its inception in the 1990s. The theory that low environmental levels of a variety of organic chemicals might be causing subtle but widespread developmental and reproductive effects on both humans and animals arose from a Wingspread conference and the book *Our Stolen Future* (Colborn et al. 1996; see also vom Saal 1995). The scientific community has responded with a reexamination of chemicals, organisms, and response end points for better characterization of the nature and extent of possible effects from low-level exposures. This has included extending and expanding existing reproductive, behavioral, and biochemical methodologies and end points and examining new ones. Various domestic and international regulatory and advisory agencies have taken a thorough and measured approach to evaluating the nature and extent of the potential problem and determining how to best incorporate findings into existing human and environmental regulations. The technical and policy challenges have proved formidable. Despite considerable effort, standard technical definitions have remained controversial and the widespread use of generally accepted testing protocols has proven to be a substantial challenge that is not yet resolved (Borgert et al. 2011b).

Largely concurrent with the development of the endocrine issue has been a debate about the importance of the provenance of toxicological testing and the possible biases of investigators. Some have argued that toxicological studies commissioned by industry are of a lesser quality or reliability because of overt biases attributed to industrial funding (e.g., Sass et al. 2005) and that scientists employed or supported in their research by an industry should be considered tainted and unsuitable for government policy or technical panels [see details and rebuttal in Barrow and Conrad (2006)]. As many investigations mandated by government receive both substantial industry funding and government staff participation, carried to the logical end, this would mean that many government scientists should also be disqualified under such a policy. As well, such an argument ignores the fact that non-industry scientists also have support-related biases that can taint their views and thereby disqualify them by the same logic. Many in the scientific community reacted strongly by recommending, in essence, “judge the science, not the scientist” (e.g., Borgert 2007; Gori 2009; Society of Toxicology 2008).

Provenance and bias became the central debate concerning a widely used chemical, bisphenol A. Relying primarily on toxicological results from several rodent studies conducted under Good Laboratory Practice (GLP), regulatory agencies in the United States and Europe concluded an adequate margin of safety exists for current human exposures to bisphenol A [i.e., European Food Safety Authority (EFSA) 2006; FDA 2008], although research and regulatory activity continues (FDA 2012). Myers et al. (2009) argued that studies conducted according to the U.S. EPA or the Organisation for Economic Co-operation and Development (OECD) GLP guidelines should not have taken priority in regulatory decision making because, in their view, the GLP process is less rigorous than the peer-review process used to determine acceptance for publication in scientific journals. Responses (Becker et al. 2009; Tyl 2009), additional claims (vom Saal and Myers 2010), and rebuttals (Becker et al. 2010; Tyl 2010) ensued.

Although central to these debates, there has been no thorough analysis of the similarities and differences between journal peer review and GLP with respect to process and objectives, the fitness for purpose of each within various scientific disciplines, and the implications thereof for regulatory/legal purposes. As an initial step, we examine the current status of the journal peer-review process and of GLP, in quality assurance and quality control of experimental data, data analysis, and interpretation for scientific publications and government reports. We then broaden the discussion to an evaluation of scientific data quality and validity in peer review, GLP, and regulatory development.

Clarifying the definition of peer review is essential. Peer review often refers to the processes used in screening articles for publication in scientific journals. However, similar approaches are used in other areas, including evaluation of research contracts/grants and private and government scientific reports, scientific policy documents, and regulatory directives. Science Advisory Board or Panel (SAB/SAP) review and assessment used by the U.S. EPA is a good example. Such non-journal peer review shares the general problems faced by journal peer review: The character, extent, and thoroughness of the evaluative process and the actions and subsequent follow-up varies widely because of the lack of a single, well-documented, broadly applicable, generally accepted peer-review scheme. Therefore, although much public debate about peer review focuses on journal peer review, our examination pertains to more broadly defined peer-review exercises.

## Evaluation of Pertinent Literature

*Peer-review history and development.* Burnham (1990), Kronic (1990), and Rennie (2003) have published good reviews on the history of scientific peer review, based largely on the biomedical experience. From its formal beginnings in the early 1700s to its modern phase of development beginning in the late 1940s, peer review exhibited two dominant characteristics. First, its nature and extent varied widely within and between journals, primarily as a function of the preference of the editor in charge. Second, peer review aided in selecting items and issues for publication based on the relevance and potential significance to the journal’s audience. Peer review and publication did not guarantee validity or correctness of the theories, data, analysis, or conclusions presented.

As scientific research increased dramatically after World War II, the number of journals expanded to accommodate it. Although journal peer review grew and developed, no consistent overall format was employed and the extent and nature of reviews continued to vary widely. By the 1980s change had begun. Long-time *British Medical Journal* editor Stephen Locke published a seminal book on peer review (Locke 1985) and in 1986 the *Journal of the American Medical Association*, at least partially in response to an article by Bailar and Patterson (1985), organized the first in an ongoing series of quadrennial conferences about research on peer review: the International Congress on Peer Review and Biomedical Publication. Although published opinions/editorials on peer review continue unabated, the study of peer review as a recognized scientific research topic stems from that time. Campanario (1998) examined the extent and diversity of research on peer review for the first two decades of this period.

A comprehensive evaluation of research literature on peer review is beyond the scope of this review, but we provide here a brief overview of prominent topics and the general turmoil. A number of recent opinions/editorials have described the general problems with peer review and illustrate the nature and extent of concerns: “Is Peer Review Broken?” (McCook 2006), “End of the Peer Review Show?” (Henderson 2010), and “I Hate Your Paper” (Akst 2010). However, these concerns are long-standing. Horrobin (1990) argued that rejection during peer review has and continues to delay and diminish new developments that challenge the status quo of established scientists who conduct the bulk of reviews. Armstrong (1997) agreed. Jefferson et al.’s (2002) focused review concluded that peer review is largely untested for its effects on scientific publication quality, primarily because of its lack of clear and consistent objectives.

In 2006, *Nature* sponsored an online Peer Review Debate that drew a number of opinion articles, including the following examples. Jefferson (2006) indicated that peer review may be the best available process but this cannot be confirmed without explicit evaluation of alternatives. Jennings (2006) argued for more quantitative measures of peer-review performance to enable better evaluation of current practices and alternatives. Lee and Bero (2006) advocated improvements in transparency and fairness policies and facilitation processes.

Despite extensive questioning of goals, objectives, and processes for journal peer review, there is no widespread movement to reduce or eliminate it, but there have been many suggestions how to improve and strengthen it. Armstrong (1997) discussed various way to change the nature of peer review from a dichotomous decision on whether to publish to a graded scheme of how and in what form to publish. Benda and Engles (2011) noted that peer review is relatively successful when viewed as an exercise in judgmental forecasting but may impede the publication of innovative work. They suggest remedies to enhance the publication of innovative work that involve changing reviewer voting and decision processes.

Cicchetti (1997) called for explicit decision criteria to aid in standardizing peer-review decision making. Others have focused on specific areas of data reporting. Kilkenny et al. (2010) advocated adopting the Animals in Research: Reporting *In Vivo* Experiments (ARRIVE) guidelines for reporting animal experimental data in health sciences, noting the success of the Consolidated Standards of Reporting Trials (CONSORT) guidelines for improving the quality and transparency of data reporting from randomized clinical trials. Borgert (2007) suggested requirements for peer-reviewed publications, including that review articles follow literature searching and selection rules adopted for systematic reviews by clinical journals and that all studies post full details online, including raw data, laboratory notebooks, and statistical algorithms. However, even this GLP-like suggestion does not address a well-known journalistic prejudice toward publishing toxicity studies that demonstrate overt effects. This applies particularly to new methods or novel uses of methods, which depend on demonstrating utility in order to gain attention and acceptance. In sum, these factors produce an underrepresentation of studies that show little or no effects, i.e., “negative results,” thus making it difficult to obtain a balanced view of many toxicological issues.

A prominent subtopic is legal/litigation use of peer review and biomedical information. The U.S. Supreme Court’s decision that although peer review can provide useful information about scientific merit, it is not an infallible metric of validity nor is it always reliable (Horrobin 2001). In Horrobin’s view, this and other recent findings require that peer review either be improved or abandoned. Henry and Conrad (2008) reviewed the issue of judging scientific work conducted for regulatory advocacy versus general scientific purposes, concluding that American judicial and administrative guidance mandate a single common evaluative scheme irrespective of the provenance, funding source, or rationale for producing the information. Boden and Ozonoff (2008) examined this issue and concluded that the various conflicts of interest are not unique to science generated specifically for litigation, but rather are general in nature and pervasive in science. They also noted that peer review should not be considered a reliable metric to judge quality and relevance.

The issue of potential conflicts of interest in legal scientific work reached such a pitch that the Society of Toxicology specifically included in its 1998 Principles for Research Priorities in Toxicology (Society of Toxicology 2008):

4. Research should be judged on the basis of scientific merit, without regard for the funding source or where the studies are conducted (e.g., academia, government, or industry).

In general, although many agree on the primacy of scientific methods and data validity in determining merit for publication and funding and that neither should be limited because of affiliation or financial interest, there is a range of viewpoints regarding the role of disclosure requirements. Some concede that disclosure of financial interests might limit participation in certain aspects of data interpretation and use (Barrow and Conrad 2006; DeAngelis and Fontarosa 2010), whereas others (Borgert 2007; Gori 2009) argue that because absolute freedom from conflict of interest is impossible, complete transparency of data and methods should be required to the exclusion of disclosures regarding finances and affiliations.

Fraud in the published literature has emerged as one of the most novel and cautionary topics of comprehensive study. Steen (2011) examined nearly 800 papers retracted from peer-reviewed biomedical journals between 2000 and 2010. Although retraction due to inadvertent errors was most common (over 70%), he also found evidence of deliberate fraud whereby authors intend to deceive but are eventually caught and forced to retract, noting that publication in prominent, high-impact journals (e.g., *Science* or *Nature*, among others) is a common characteristic of such work. One prominent example from the endocrine disruption field was published in *Science* (Arnold et al. 1996). Despite its retraction the following year (McLachlan 1997) and eventual designation as fraud [Department of Health and Human Services (DHHS) 2001], the discussion and concern initially generated by this purported “groundbreaking” paper figured significantly in regulatory decisions and public policy.

Even the vigorous prepublication peer-review processes of high-impact journals cannot be expected to identify all deliberate attempts at fraud and deception. The U.S. Office of Management and Budget (OMB) concurred that although journal peer review is valuable, there are many documented cases of flawed or falsified data being published (OMB 2002). Post-publication attempts at replication are typically responsible for identifying untrustworthy methods or deliberate deception, which highlights that journal peer review is not an entirely reliable metric of data quality or scientifically sound conclusions.

In summary, many problems have been identified with the journal peer-review process, but the proposed solutions favor revision rather than replacement. Importantly, the purpose of peer review is not to assure the quality of study designs or methods of data collection or analysis, or that interpretations are unequivocally supported by the data. Rather, the purpose of peer review is to help ensure that published articles are worthy of consideration and debate. To that end, peer review considers whether articles address timely topics, are interesting and relevant to the target audience, whether details of methodology and results are sufficiently well described to allow replication, and that conclusions are supportable (not uniquely or unequivocally proved) from the data presented. Given this limited scope, one should not expect journal peer review to detect fraud, misconduct, or even all degrees of biased reporting and interpretation. What can be encouraged, however, is adopting a more standardized process for peer review that focuses on full and transparent reporting of not only data generation and analysis but also on the manuscript review process itself. Not only would this promote fairness for novel findings and more readily identify misconduct or bias, it would facilitate post-publication evaluation for other uses, such as regulation and litigation.

*GLP history and development.* The historical development of GLP is well documented (e.g., OECD 1998; World Health Organization 2001). Briefly, the U.S. FDA responded to various issues and problems with experimental conduct and reporting in new drug registration submissions by proposing a GLP regulation in 1976 that became effective as a Final Rule in 1978. This focused on improving the quality of preclinical drug safety data by mandating specific experimental conduct and reporting protocols. GLP regulations from the U.S. EPA followed, with Final Rules effective in 1983. GLP for international forums was addressed by OECD Principles of GLP and a directive on Mutual Acceptance of Data in the Assessment of Chemicals (OECD 1981a, 1981b). OECD GLP guidance has since been revised and expanded to a series of 15 guidance documents on various issues and aspects (OECD 2011) that have been formally adopted in various OECD jurisdictions [e.g., European Union (EU) 2004]. OECD GLP has always focused clearly and explicitly on quality assurance, quality control (QA/QC) (OECD 1998):

Good Laboratory Practice (GLP) is a quality system concerned with the organizational process and the conditions under which non-clinical health and environmental safety studies are planned, performed, monitored, recorded, archived and reported.

GLP also has a long-term objective: mutually acceptable data (OECD 1981b). Mutually acceptable data ensures that sufficient experimental data is collected and reported so different jurisdictions can use GLP-conducted studies to fulfill the requirements of local regulations. This limits potential technical trade barriers, reduces overall costs of providing chemical regulatory data, and reduces animal use in regulatory activities.

Lest GLP be viewed as an international standard that has been implemented and enforced uniformly across the globe, Helder (2008) has noted that implementation of GLP inspections of test facilities varies among OECD members despite the common objective of certifying that data generated within these facilities are reliable and can be used for the assessment of chemical safety in all jurisdictions. Similarly, Huntsinger (2008) found that the modest but significant differences between GLP implementations by the U.S. EPA, FDA, and OECD do not affect the data quality or integrity, but increasing harmonization is an important ongoing goal.

In addition to the broader direction provided by high-level OECD, U.S. EPA, and FDA documentation, detailed project-specific GLP guidance has also been developed. The U.S. Fish and Wildlife Service uses GLP-based protocols in non-human drug testing [Aquatic Animal Drug Approval Partnership (AADAP) 2006a, 2006b]. Harmonized guidance for GLP compliance promotes consistency among EU member states in which data are generated (EU 2004). The European Chemicals Agency (ECHA 2008) seeks comprehensive GLP compliance for relevance and reliability and requires a relevance checklist and answers to GLP-like reliability questions on data availability and method description.

In summary, GLP originated in the United States but quickly became internationally recognized, through the auspices of the OECD, as quality assurance/control processes for ensuring that experimental scientific protocols and data reporting on chemical testing for regulatory purposes are conducted in a thorough and standardized manner, such that the information generated is acceptable for various activities in multiple jurisdictions. The ongoing development of GLP guidance continues to have a dramatic influence on how scientific research is conducted, reported, and used for regulatory purposes.

## Discussion

The historical background on peer review, GLP, and related scientific information quality initiatives and practices (e.g., Batterman et al. 1999; Burnham 1990; Kronic 1990; Rennie 2003) provide context for addressing two key issues identified earlier: researcher bias and data validity. Myers et al. (2009) opined that GLP-compliance should not be the gold standard for scientific information used in regulatory activities, but rather that scientific reports that have been through a journal peer-review process should be. The heart of their argument is two-fold. First, they assert that scientific peer review is a coherent, consistent evaluative process providing quality control for data generation, analysis, and reporting, thereby providing a basis for establishing relative merit of the information and the strength of the conclusions. Second, they assert that GLP is not a peer-review process and provides inadequate or inferior quality assurance/control; therefore, information and conclusions obtained under GLP are inferior. These aspects of their argument are addressed consecutively below.

It is difficult to extract from the extensive body of work and commentary published over the last 25–30 years that scientific journal peer review is a coherent, consistent, reliable, evaluative procedure. Based on the overview presented earlier, the opposite conclusion may be more accurate. Unlike GLP, which, as a formal QA/QC process, has specific written goals and guidance that are reviewed and updated periodically, peer review as conducted by scientific journals is characterized by varying policies and processes. Each journal determines reviewer selection procedures, instructions to reviewers if any, and the process by which manuscripts are accepted for publication. Approaches vary between and within journals, with passage of time, and with changes in editorship. This diversity of approaches and thoroughness precludes a coherent, consistent process for evaluating manuscripts for peer-reviewed journal publication. In addition, contrary to the assertions of Myers et al. (2009), systematic analyses of clinical research have found no consistent association between funding source and data reporting quality among top journals (e.g., Kaiser et al. 2011).

The peer-reviewer training package of the *British Medical Journal* (BMJ 2004) is particularly noteworthy among early examples of formal, documented guidance for journal peer review, containing background material, clear process objectives, examples of good reviews, and detailed guidance on how to conduct a review for this journal. Nonetheless, ascertaining fundamental issues of data quality and integrity and the scientific soundness of the interpretations can be difficult because nearly all journal peer-review evaluations proceed without access to the underlying data or, often, to detailed information regarding experimental methods. As publishers winnow articles to expand readership and reduce printing costs, they inadvertently restrict the information reviewers and readers need to properly evaluate the science.

The second component of Myers et al.’s (2009) argument is that GLP is not a peer-review process and is inferior to it. This is true only in the sense that GLP does not require relatively unstructured, confidential comments from a few scientists knowledgeable in the general research area addressed by the paper under review. Unlike journal peer review, GLP gives clear and detailed *a priori* guidance to practitioners concerning what information to collect and how to collect and report it. Current OECD GLP represents the collective guidance of hundreds, if not thousands, of scientific and technical experts who are peers of those who use the GLP process. GLP guidance itself is periodically reviewed, revised, enhanced, and expanded.

Because GLP is often applied to guideline toxicity studies required by regulatory mandates, it is frequently misconstrued as synonymous with guideline studies. The criticism is that guideline studies, and hence GLP by erroneous association, may not incorporate the most recent advancements in a particular field. Irrespective of whether they are state-of-the-art, the relevance and reliability of guideline studies are documented by a defined process, and many have been subjected to formal validation exercises, including peer-reviewed ring-testing in multiple laboratories with subsequent peer review of the data and analysis. Studies published in scientific journals often employ methods too new to have undergone such testing, reflective of their different emphasis. Notwithstanding, GLP can and is often applied to novel exploratory research studies.

In practice, GLP is a framework for experimental planning and a formal QA/QC process requiring detailed documentation of what was done and how. Compliance confirmation by QA/QC officers is required and provides a measure of reliability and validity that the chosen design was followed. Furthermore, GLP projects and facilities are subject to compliance audits by formally trained personnel. This is not to imply that GLP guarantees correct interpretation, analysis, and conclusions of experimental data or that the most probative and cutting edge techniques are always employed. GLP does not address all aspects of scientific validity any more than does journal peer review. It does, however, ensure secondary validity of the data to the greatest extent achievable. As a result, GLP enables thorough reexamination and reevaluation of the raw data, either to check the original interpretation or to carry out novel analyses. For example, a reanalysis of Pinter et al. (1990) by the Atrazine SAP determined that the male mammary tumors present at high dose occurred in rats that lived significantly longer than controls [Federal Insecticide, Fungicide, and Rodenticide Act (FIFRA) SAP 2000]. The original peer review failed to discern that tumors were due to aging rather than to atrazine. This and other flaws in study design and data analysis were later acknowledged by the SAP and U.S. EPA (2000). Such reanalysis was possible only because of reporting requirements involving QA/QC assurance, preservation, and availability of raw data.

Thus, arguments over the superiority of journal peer review versus GLP compare dissimilar entities designed for different purposes. GLP serves certain regulatory purposes exceedingly well, and undoubtedly better than journal peer review processes could. There is also legitimate concern that regulatory review should include considerations prominent in journal peer review that are not included in GLP, but this does not obviate the clear benefits of GLP. Resolving the controversy may instead require enhancing both processes.

*Convergence of GLP and peer review.* GLP and peer review are both useful to scientific reporting and evaluation. On one hand, the overall objective of peer review is to ensure that published articles are worthy of consideration and debate by the scientific community, providing new, relevant, interesting, readily comprehensible material in various fields of interest. On the other hand, the overall objective of GLP is to ensure thorough, consistent, and detailed reporting of all aspects of experimental investigations so that reanalysis and reevaluation are readily possible. Despite the rigor that GLP brings to data collection and reporting, there is no impetus to require it for all scientific investigations (e.g., Miller et al. 1999). Some argue that the additional costs of strict GLP compliance would be prohibitive, especially for academic research. However, the increased transparency would aid peer reviewers in evaluating overall merit for publication, as well as facilitate their detecting inadvertent errors and deliberate fraud.

It would appear logical for GLP and peer review to converge in some aspects while maintaining their differing primary objectives. Such convergence is evident, largely from the peer-review field. Some journals now request additional data reporting and many provide options for electronic publication of supplemental material. The explicit peer-review training and documented guidance of the *British Medical Journal* has been noticed. Some journals are experimenting with innovative peer-review approaches, including better guidance, open or non-anonymous reviewing, and variations on limited prepublication screening with subsequent open commentary.

Regulatory authorities are also becoming more interested in ensuring clearer communication of scientific concepts and conclusions, as well as increased transparency of data. Subsequent to the 2001 enactment of the Information Quality Act in the United States, the OMB released its “Guidelines for Ensuring and Maximizing the Quality, Objectivity, Utility, and Integrity of Information Disseminated by Federal Agencies” (OMB 2002). More recent guidance on peer review and risk assessment continues to emphasize data quality and clear communication (OMB 2004, 2007). Other agencies, such as the U.S. EPA and U.S. Fish and Wildlife Service, as well as those falling under the DHHS [e.g., the FDA and National Institutes of Health (NIH)], have developed mandate- and facility-specific guidance tailored to their activities (AADAP 2006a, 2006b; Birnbaum and Culpepper 1999; DHHS 2006; U.S. EPA 2006). The Registration, Evaluation, Authorisation and Restriction of Chemical substances (REACH) process in the EU also has guidance addressing relevance, reliability, and adequacy of data (ECHA 2008).

Guidance for risk assessment review sets requirements for methodology reporting and data availability and quality that helps bridge the GLP/peer-review chasm (OMB 2007). In Canada, the *Framework for Science and Technology Advice* (Government of Canada 2000) states clear government-mandated quality-based principles for conducting and evaluating both scientific information and decision-making processes used in regulations protecting human and environmental health. However, Forristal et al. (2008) noted that specific operational frameworks are not fully available for applying these principles in generating chemical risk assessments.

Finally, but conspicuously absent from the discussion by Myers et al. (2009), is the fact that regulatory agencies conduct their own case-specific peer review of all data from all sources pertinent to the regulatory guidance being developed. As GLP-based reports are explicitly designed to facilitate pre- and post-publication review, it should not be surprising that, because of strict reporting requirements, they are more readily reevaluated for data quality. Thus, the confidence placed in data from GLP studies is often justifiably greater than in data from peer-reviewed journal papers, where thorough data reexamination is often limited by a lack of reporting detail. Methodological convergence between journal peer review, GLP, and regulatory decision making will certainly continue as all three have similar objectives for data transparency and quality. Convergence will have a number of benefits both within and between these three evaluative activities.

*Validity: data quality, overall study quality, relevance.* Borgert et al. (2011b) has described three tiers of scientific validity. To be considered established scientific facts, scientific data must minimally conform to three tenets underpinning the basic language of science that enables trustworthy measurement of the natural world (Gori 2009). This might be called “primary validity” of the data. First, the identity and authenticity of scientific measurements must be verifiable within a defined range of precision. Second, measurements and observations must not be confounded by extraneous factors and influences known to corrupt their accuracy and precision. Third, the measurements and observations must be replicable in independent hands. These three tenets are undeniable and agreed upon as the minimum requirements for valid regulatory science in the United States (U.S. Congress 2010; U.S. House of Representatives 2010). We believe they are also sufficiently unambiguous to provide the primary standard against which all data should be judged. Establishing the reliability of data also requires transparency and thoroughness of data reporting (Klimisch et al. 1997), which constitute “secondary validity” of the data. The overall relevance of the study and merit for publication might be termed “tertiary validity” of scientific data. These three tiers of scientific validity (Borgert et al. 2011a) encompass the necessary elements of scientific data evaluation. GLP and peer review incorporate important aspects of validity, such as precision and regulatory relevance, but neither fully addresses all three tiers in an explicit manner.

So-called “weight of evidence” (WoE) evaluations are often undertaken to examine, prioritize and integrate results for different types of studies used to reach regulatory decisions. To achieve the desired goal of clearly identifying overall study quality and establishing relative merit for input into regulatory decision making, explicit processes specific to the hypotheses or questions at hand (Borgert et al. 2011a) are needed. Klimisch et al. (1997) defined reliability, relevance, and adequacy in terms appropriate for such a task, and they also defined four categories of study/data reliability: reliable without restrictions, reliable with restrictions, not reliable, and not assignable. Schneider et al. (2009) operationalized the use of the Klimisch reliability categories by developing an evaluative tool with uniform, objective category criteria enabling scientifically sound evaluations and assignment of relative merit weighting to toxicological studies and data. This evaluative tool can be used with all studies, both GLP and non-GLP, of interest in a given situation. A similar tool was developed for assessing data from ecotoxicology studies (Hobbs et al. 2005). In both cases, a set of questions was developed to guide the evaluation in rating the scientific rigor of both published and unpublished data to help harmonize reviews and increase transparency.

Schneider et al. (2009) and Hobbs et al. (2005) identified variability in peer-review assessment of reporting and interpretive quality among publications evaluated in their studies. Hobbs et al. (2005) noted that conflicting peer evaluations occur for a number of reasons, including failure to find data in the report and interpretative disagreement. For example, Hobbs et al. (2005) recounted that while outside reviewers all thought that temperature was measured in one of the studies they evaluated, the study stated only that a chamber temperature was set, but no measured readings were presented. Schneider et al. (2009) also found that reviewers scored information differently depending upon their interpretation of the questions asked in the evaluation scheme.

If Schneider et al. (2009) and Hobbs et al. (2005) represent peer review of peer-reviewed publications, the variability in reassessment of manuscript quality and completeness points to the problems in journal peer review. If reviewers cannot agree on answers to specific questions about a manuscript that has already passed peer review, how thorough and dependable could their review be when no specific questions or guidance are provided? At a minimum, a checklist as advocated by Schneider et al. (2009) and Hobbs et al. (2005) would help guide journal reviews in a more GLP-like manner.

However, no matter how high the quality score achieved, any particular study may not be the most useful for specific decision making. One additional evaluation step is neither present nor feasible in either journal peer review or GLP. Although data relevance is vital to study relevance, of necessity, in WoE it is evaluated within a general relevance construct rather than in a case-specific context. The concept of specific relevance, however, is well described by U.S. EPA (2006):

DQA [data quality assessment] is built on a fundamental premise: data quality is meaningful only when it relates to the intended use of the data. Data quality does not exist in a vacuum, a reviewer needs to know in what context a data set is to be used in order to establish a relevant yardstick for judging whether or not the data is acceptable.

Although judgments about relevance and adequacy in regulatory applications have case-specific aspects, it should be possible to develop general categories and criteria, similar to those proposed by Schneider et al. (2009) and Hobbs et al. (2005), which would better assist in determining specific relevance. This would provide more transparent and effective weight-of-evidence schemes for evaluating the relative merit of toxicological studies/data for regulatory decision making. Such an approach obviates the need for any peer review versus GLP arguments and places emphasis on the key issue facing regulatory decision makers: establishing the reliability, adequacy, and relevance of all available toxicological information on a given issue. A well-documented, generally accepted weight-of-evidence scheme designed to evaluate both journal peer-reviewed and GLP information for regulatory activities would also aid in updating schemes for determining relative merit and general validity in journal peer-review and GLP activities. The challenge is balancing data validity and specific relevance.

## Proposed WoE Scheme

Background information on WoE and overarching scientific principles that apply generally and to endocrine disruptor screening (U.S. EPA 2011), as well as an example of a hypothesis-driven WoE approach derived for a specific regulatory purpose (Borgert et al. 2011a), will not be repeated here in detail; however, some salient information will be referred to as needed. Fundamental principles can be broadened to develop WoE frameworks generally appropriate for regulatory peer review. Specific components can be handled flexibly to account for different regulatory goals and applications. To do this, “weight” and “evidence” must be clearly defined for credibility and transparency.

As noted in the previous section, scientific evidence has been defined (Gori 2009) according to primary, secondary, and tertiary validity of the data (Borgert et al. 2011a). These concepts are well accepted, relatively firm, and when combined with recommendations for transparent reporting of literature search and selection procedures, can be used to evaluate all toxicological studies. As toxicological data and analyses are often applied to situations and circumstances unforeseen by the primary investigators, the user will need to consider the original intent and the newly proposed application. If possible, hypotheses to be tested by the new application should be explicitly defined. The new application would guide the literature search and selection process, which should be formulated and documented before conducting the WoE analysis. Primary and secondary validity can be assessed within the context of original intent as the soundness of measurements and reporting quality are unlikely to change with a new application. Tertiary validity will often need to be evaluated within both contexts because a study design probative for its original purpose may fail to include components critical to a new application. With rare exception, however, a study too weak for its original purpose is unlikely to gain probative strength for another.

“Weight,” on the other hand, implies that a different value or importance is assigned to different data, and thus “weight” must be defined more contextually than “evidence.” Weighting is the step where the user must carefully consider the intended regulatory application rather than the investigator’s original focus. Ideally, the purposes would coincide and weight could be assigned quantitatively (Borgert et al. 2011a), accounting for factors such as predictive power and false positive/negative detection rates. Whereas original and regulatory purposes may differ and quantitative groundings are often unattainable, flexibility is essential for broad applicability across varied regulatory activities. Flexibility might allow for the explicit inclusion of provisions to offset publication bias against negative toxicological data, which can be particularly problematic for the newest methodologies or novel applications of existing methods. Nonetheless, two factors are critical for a successful and generally acceptable WoE scheme: *a*) the process used to weight various types of data, including its literature basis, must be transparently and clearly articulated, and *b*) the weightings themselves must be derived *a priori* and applied consistently.

A full complement of examples is beyond the scope of this review, but guidance on evaluating specific data relevance for regulatory uses (U.S. EPA 2006) suggests processes for identifying and weighting data for specific applications. In our view, however, a credible WoE evaluation scheme must include specific criteria and steps to be followed in addition to describing general principles. Irrespective of whether data are prioritized according to species, route of exposure, assay protocol, reagent grade, pharmacokinetic assessment of dose, field versus laboratory, or any other parameter, each weighting should be justified with a clear and fully referenced explanation. It is within this process of weighting information for a particular purpose that the arguments over GLP versus journal peer review dissolve. For regulatory applications requiring data reanalysis, GLP-like characteristics may be of utmost importance. For other purposes, data derived using the most sensitive and updated analytical techniques may take precedence. Data priorities should be defined by fitness for purpose rather than by predetermined preferences for source and provenance.

Our proposed WoE scheme for regulatory peer review comprises the following six general steps:

Define the specific regulatory application and its goals, including explicit hypotheses to test, if possible.
Define priorities for weighting different types of data or study characteristics and develop a referenced rationale for general weightings based on the regulatory application.
Systematically search, review, and select data relevant to the new application and hypotheses.
Evaluate the primary, secondary, and tertiary validity of each selected study based on its original intent, and evaluate the tertiary validity of each study based on the new application.
Combine data quality evaluations with data weightings according to a predefined algorithm to produce a WoE score for each study or datum.
Integrate WoE scores for all pertinent data and develop an overall WoE narrative that describes all judgments and conclusions derived from the WoE evaluation process, including key assumptions, uncertainties, and any adjustments or refinements in weighting factors required subsequent to their initial formulation.

## Summary

We have reviewed the background and explored the basis for improving and expediting environmental decision making on several fronts—peer review and GLP as well as the development of regulations—by arguing for coordinating common elements where possible and by pointing out where convergence is occurring. A key aspect for achieving such a goal is a broadly applicable, generally accepted WoE decision-making framework. What is needed, rather than the current, task-specific approach to decision making, is a general WoE framework for informing and guiding various regulatory decision-making tasks. Although there are regulation-specific issues, common WoE principles would facilitate intercommunication and efficiency. In the interim, we note the convergence where journal peer review is incorporating more data transparency and reporting aspects, similar to concepts more fully realized in GLP. This convergence should improve assessments both within and between various evaluation schemes and ultimately, improve and expedite peer review, use of GLP, and regulatory decision making.

## Conclusions

Evaluating the quality of scientific information used in regulatory decision making requires that judgments be made about data production processes, data reporting, analysis, and interpretation methods and data applicability relative to the goals of the decision-making activity in question. Ultimately, these judgments require that data validity and specific relevance be considered, evaluated objectively and transparently, and adjudicated consistently. Journal peer review, GLP, and regulatory rule development share common interests in validity evaluation. However, as they differ in their decision-making goals, process implementation and resultant outcomes are not fully comparable.

Journal peer review achieves valuable screening/​prioritization in the process of bringing new, relevant, and interesting data and issues to the attention of scientists in a readily comprehensible manner. However, it is currently not a reliable process for establishing data quality, nor does it represent an unequivocal metric for establishing relative merit of data or interpretation and conclusions drawn from those data.

GLP is best at establishing data quality, especially as the mandated documentation requirements allow for thorough, independent reanalysis and reinterpretation. It is not foolproof, nor does it provide an unequivocal metric for establishing general validity or relative merit of interpretation and conclusions. The focus of GLP is often, but not exclusively, on the execution of approved guideline-based studies such as toxicity assays required by regulatory mandate to probe a specific biological response. Some guideline assays have been subjected to a validation process, including ring-testing in multiple laboratories, wherein their predictive capacity and relevance have been determined. As such, GLP represents a legitimate selection or weighting criterion for data used in regulatory decision making.

Neither peer review nor GLP are, on their own, mechanisms to determine relative merit, general validity, or scientific soundness of data interpretation and subsequent conclusions drawn from that interpretation. Nor are they intended to be. No single gold standard evaluative process with broadly acceptable, generally applicable decision criteria exists.

Peer review is moving toward revisions and improvements in several areas. In particular, clearer documented evaluation guidance and processes are being employed by some journals. As well, many journals are encouraging the publication of supplemental material that provides more details of methods and results than appears in the main publication. These changes move journal peer-review methods closer to approaches used by GLP.

Both peer review and GLP provide useful insights into data and results from scientific studies, but neither alone is sufficient for establishing relative merit and scientific soundness of the research. The solution lies in developing a well-documented, generally accepted weight-of-evidence scheme that is designed to compare, contrast, and evaluate both peer-reviewed and GLP information and to determine relative merit and general validity. This proposed scheme could readily feed into regulatory decision-making processes where case-specific validity judgments, necessary for effective decision making, are made using such data quality evaluations.
